# Application of Functionally Graded Shell Lattice as Infill in Additive Manufacturing

**DOI:** 10.3390/ma16124401

**Published:** 2023-06-15

**Authors:** Slawomir Kedziora, Thierry Decker, Elvin Museyibov

**Affiliations:** Faculty of Science, Technology and Medicine, University of Luxembourg, 6 rue Coudenhove-Kalergi, L-1359 Luxembourg, Luxembourg; thierry.decker@uni.lu (T.D.); emuseyibov94@gmail.com (E.M.)

**Keywords:** functionally graded lattice structure, infill, design exploration, finite element method, bicycle crank arm, additive manufacturing

## Abstract

The significance of lightweight designs has become increasingly paramount due to the growing demand for sustainability. Consequently, this study aims to demonstrate the potential of utilising a functionally graded lattice as an infill structure in designing an additively manufactured bicycle crank arm to achieve construction lightness. The authors seek to determine whether functionally graded lattice structures can be effectively implemented and explore their potential real-world applications. Two aspects determine their realisations: the lack of adequate design and analysis methods and the limitations of existing additive manufacturing technology. To this end, the authors employed a relatively simple crank arm and design exploration methods for structural analysis. This approach facilitated the efficient identification of the optimal solution. A prototype was subsequently developed using fused filament fabrication for metals, enabling the production of a crank arm with the optimised infill. As a result, the authors developed a lightweight and manufacturable crank arm showing a new design and analysis method implementable in similar additively manufactured elements. The percentage increase of a stiffness-to-mass ratio of 109.6% was achieved compared to the initial design. The findings suggest that the functionally graded infill based on the lattice shell improves structural lightness and can be manufactured.

## 1. Introduction

**Figure 1:**
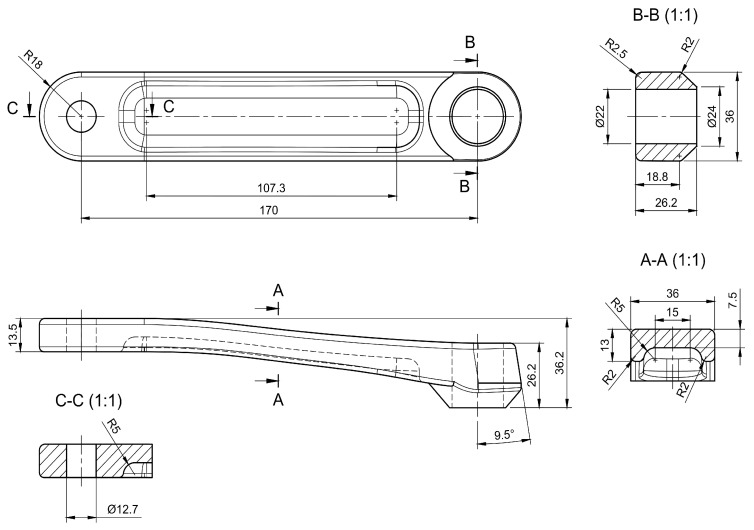
CAD model of crank arm created based on Shimano FC-R450/453 crank arm.

The significance of lightweight designs has become increasingly paramount due to the growing demand for sustainability. Consequently, this study aims to demonstrate the potential of utilising a functionally graded lattice as an infill structure in designing an additively manufactured bicycle crank arm to achieve construction lightness. The bicycle crank arm (Figure 1) is a part where lightness is essential and self-evident since the vehicle is powered by human muscle power, and minimal energy input is desired.

With this publication, the authors aim to demonstrate that designing and manufacturing parts with optimised internal infills as a functionally graded shell structure using metal Fused Filament Fabrication (FFF) technology is possible. Such a solution does not currently exist, and its creation could contribute to the further development of this new printing technology. In addition, the authors want to prove that this can significantly improve the structural lightness of the fabricated parts. Thus, the authors’ efforts were principally targeted at answering the following questions:

- How to represent the geometry of a 3D printed object with a functionally graded infill efficiently?
- How to identify an optimal design among many viable options, and how to include manufacturing constraints?
- Does the developed part fulfil functional requirements?
- How to validate its structural performance theoretically?
- Is manufacturing the part with a functionally graded infill feasible with the metal FFF technology?

To answer these questions accurately, the authors conducted the entire process of designing and analysing a new part for specific loads, including manufacturing constraints, and then created a prototype. In order to achieve this, three elements must be in place: practical design tools, a structural analysis methodology and a reliable manufacturing method. These three components form an inseparable process that aims to create a lightweight crank arm with an optimised functionally graded shell lattice as the infill.

Thus, this project’s primary outcome is a robust design and simulation process for additive manufacturing targeting the metal FFF method with the infill as functionally graded lattices. The metal FFF is often called the Material Extrusion Method (MEX). It characterises that wire-shaped metal-containing plastic (filament) is plasticised in a nozzle and selectively put locally layer by layer, building a 3D part, which is washed and sintered, receiving a finished metal part [1]. A main benefit of the metal FFF technology in the project’s context goal is the possibility of creating parts without adding powder-release channels for loose powder from internal cavities, as required in powder bed fusion processes.

Any internal structure of the 3D printed objects, called an infill, was an optimised item. The infill usually has regular structures determined in slicing software as a total volume percentage. The infill structures determine the mechanical properties of the printed parts and impact the printing process [2]. More material in the infill leads to a more robust but heavier object and extends the print time.

As the infill, very promising structures are shell-based lattices because of their stiffness, strength, and printability, supported by the literature review. Therefore, the authors implemented one in the crank arm. Many researchers have been working on the mechanical responses of the lattice structures in recent years [3,4,5,6,7,8,9]. However, their projects have focused mostly on specimens or simple parts with basic load cases—compression and/or tension. Unfortunately, attempts were missing to show how to benefit from those structures as the infill in functional parts with complex stress states that cannot be simplified to only tension or compression. The shown work fills this gap by demonstrating a real part’s investigation/optimisation process. As a result, the authors used a Gyroid shell lattice for the internal functionally graded infill, mainly considering its stress concentration-free characteristic, sufficient stiffness, and printability.

It should also be noted that there are solutions where an open lattice is a part of the structure. The paper’s authors [10] show this structure with the example of a suspension arm analysed using lattice structure optimisation. They concluded that strength improvement and weight reduction could be reached using various types of lattice structure and topology optimisation.

Further, the article text presents the background research to explain the applied methodology and to present the status quo. Next, in the Section 2, the analysis method is described with the level of detail required to reproduce the analyses. The authors also present assumptions that were made, models, and material properties, giving all data. Subsequently, in the Section 3 and Section 4, obtained results and comparison with existing designs highlight the achieved improvements. Findings are discussed, focusing on structural performance, part lightness, and the developed method. Lastly, the authors summarised the work in the Section 5 and proposed further directions for the planned investigations.

### 1.1. Lightweight Constructions

Lightweight constructions are characterised by low material density and unique methods of shaping and specific manufacturing, and all are implemented to achieve maximum stiffness at a given weight and strength. Lightweight and ultra-light structures are critical due to reducing machines’ energy consumption and sustainability. The main obstacle to the increased use of lightweight parts is cost pressure in individual industrial sectors [11,12,13]. However, the future of creating new lightweight products looks very encouraging because of the increasing demand for such products in the last few years. This trend is fostered by the rapidly developing new additive manufacturing production technology.

Understanding natural structures is fundamental to designing lightweight designs effectively. In nature, lightweight objects have been ubiquitous for millions of years and have improved over time due to the evolutionary process. Natural creative processes based on the efficient use of resources have built structures unrivalled for us in many ways. For instance, in the bones of living beings, there is a mechanism causing the bone structure to grow primarily in locations with high cycling strain and disappear where the strain is low [14]—that remodelling occurs in bone mass and architecture because of stimuli obtained from its mechanical environment. That mechanism creates structures with different densities depending on external loads. Plants have also developed lightweight structures through natural evolution; bamboo is an excellent example. The bamboo stem has an optimal solution for strength and stability in specific environmental conditions. Bamboo is a nature-designed functionally graded material because the fibres’ volume fraction increases radially from the inner to the bamboo stem’s outer surface [15].

However, engineers can achieve structural lightness through a variety of techniques: lightweight and high-strength materials, new design and analysis methods, and modified or new manufacturing technology. The decision on which strategy to use depends on various aspects of the specific development needs. This project aimed to mimic nature in design (biomimicry) by using exploration methods in the design phase and the metal FFF in manufacturing.

### 1.2. Additive Manufacturing

Additive Manufacturing (AM) processes give engineers the most design freedom and produce physical objects from a computer model with few design restrictions, depositing layer-by-layer material systematically until the whole object is created without any shaping tools. AM technologies have several methods: binder jetting, directed energy deposition, material extrusion, material jetting, powder bed fusion, sheet lamination, and vat polymerisation. A brief review of the entire AM technology can be found in the article [16].

A specific metal AM technology was chosen for the project: metal fused filament fabrication (FFF). This technology allows us to maximise benefits from the optimised internal lattice structure. The primary advantage of this approach is that it facilitates the generation of intricate infill patterns without necessitating the incorporation of power-release channels. This obviates the need to extract unsintered, loose powder from internal cavities created by the internal structures. Most significantly, this method is highly cost-competitive [1].

This process belongs to the extrusion method, and it is based on a standard FFF method for polymers, also known as Fused Deposition Modelling (FDM). In this method, metal powder bound in plastic is printed layer by layer into the object’s shape. The object size is scaled up to compensate for shrinkage during sintering. At that stage, the printed part is soft and brittle (green part). The printed part can then go through a washing stage to remove the binder or directly to the sintering process. Finally, the object is sintered in a furnace to fuse the metal powder into solid metal. The metal part is created as the process’s results and can be post-processed like any other metal part. The technology is still in development, and some issues exist, such as print size restriction, support structure requirements, available materials and the printed object strength [17].

### 1.3. Infill as Functionally Graded Structure

Functionally Graded Materials (FGMs) are materials endowed with spatial variations in the physical properties and chemical compositions, which act as functional qualities. The FGM idea was proposed in the 1980s by Japanese researchers [18], who developed a new class of composite materials for aerospace applications dealing with very high-temperature gradients. FGMs can be artificially created or formed by natural evolution. The examples of bones and bamboo show natural functionally graded materials [15]. Designed FGMs are used nowadays in different industries: machinery [19], medicine (implants) [20], aerospace [19], etc. FGMs are very often cellular materials, which include foams and lattices. Their cellular characteristic can be observed differently depending on the scale: nano (1 A–1 μm), micro (0.1 μm–1 mm), and meso (0.1–10 mm). In the presented work, the internal structure—the infill has functionally graded properties through the variable thickness. However, their variations are on a millimetres scale, and therefore it would be better to name them Functionally Graded Structures (FGS) [7] instead of FGMs. Using mesoscale is determined by the current capability of metal (FFF) technology.

Designing functionally graded structures requires complex optimisation methods with considerable effort because of a high dimensionality of a model representation causing high computational costs [21]. However, as shown in [21,22], it is feasible to design and create functionally graded materials efficiently. The authors showed in the article [21] the novel general structural optimisation method—the particle swarm optimisation (PSO), a nature-inspired optimiser, for parts with functionally graded material properties. Their numerical simulations presented that the proposed approach is practical, flexible, and computationally efficient for FGM optimisation problems. The developed optimiser outperformed a classical mathematical programming-based optimiser, and the proposed approach is applicable to FGM objects with 2D and 3D geometries and any heterogeneous feature tree structure as a model of FGM variations. In the book [22], the authors give an excellent overview of FGM detailed material mechanics, modelling, applications, and manufacturing methods.

In the research area of FGS, there is a recent article [23] in which the authors propose a new approach for generating bone-like porous structures. The paper proposes a novel formulation for generating porous structures based on structural optimisation considering the optimum design from a mechanical perspective and analysing it through detailed parameter studies. The method is an extended voxel-wise topology optimisation algorithm, which maximises the mechanical stiffness by optimising the distribution of a given amount of material in a specified design domain under a given set of external loads. The authors successfully show the optimised 2D and 3D infill, including a manufacturing constraint—a minimum feature size. The authors did not focus on manufacturing constraints such as overhangs, avoidance, and closed voids containing unsintered powder occurring in the process of Selective Laser Sintering (SLS) for plastic materials. The work showed that it is possible to generate the optimised infill for bone-like structures in conjunction with SLS printing technology.

The next exciting work related to the presented project is the article [2], wherein the authors investigated numerically and tested 2D and 3D biologically inspired infill patterns in cylindrical tubes. The infills were defined in a geometrical form of Gyroid, Schwarz D, and Schwarz P surfaces. The authors found that 2D (honeycomb) infills, such as rectangular or hexagonal lattices, are unsuitable for structural applications with complex 3D geometry because of the resulting imposed anisotropy. In the case of loaded and supported classical structural elements (when a 2D infill pattern is aligned with the principal stress orientation), 2D infills may outperform 3D infills. The authors also proposed optimising the local infill density based on the actual stress fields and performance requirements, modifying an infill wall thickness to achieve uniformly distributed stress. That idea was similarly realised in the presented work, showing that it is feasible for metal FFF printing. Inspired by these articles, we used a general, robust and affordable method of analysis of the functionally graded infill of the crank arm, namely design exploration.

### 1.4. Design Exploration

Design Exploration (DE) or Design Space Exploration (DSE) [24] is a computer-assisted approach to arriving at an optimal design solution. Design space exploration must be performed carefully because a large complex system may admit millions, sometimes billions, of design alternatives. Therefore, a manual approach to DSE is unachievable. It comprises the following elements: a representation of design space, an analysis equipped with computer-assisted techniques for discovering potential design candidates, and an exploration method for exploring many design candidates [25]. Therefore, various procedures are included in the design exploration; they are the design of experiments (representation), response surface modelling with optimisation (analysis), and data mining (exploration method). These tools enable us to explore, understand, and improve the design before a conceptual phase of product development. The authors used the DE for the presented problem to obtain a set of optimal designs. The implementation of DE is described fully in Section 2.6 to avoid limitations here.

### 1.5. Lattice Types

Various researchers have investigated different types of lattices/cellular materials and their applications, including structural components [26], energy absorption [27], heat exchange [28], and biomaterials [20]. In the context of application in lightweight structures, it is known that the best option for lightweight constructions is to use stretching-governed lattice structures (strut-lattice types) based on Maxwell’s stability criterion [29,30]. The necessary condition for strut-lattice systems to be stretching-dominated lies within the connectivity that the structure’s unit cell satisfies Maxwell’s criterion for static determinacy [29]. The stretching-governed structures are expected to be about three times as strong as the bending-governed ones [29]. As shown in [29], the deformation of most foams, whether open or closed cells, is bending-dominated.

The presented investigation work was preceded by an analysis of strut-lattice implementation in the identical crank arm, as presented in the article [31]. This article shows the practical application of beam-based functionally graded lattice structures using a blend of manual adjustments and numerical optimisation methods similar to those presented in the current work. Theoretically, the conclusions were that implementing the functionally graded lattice structures (strut-lattice) in the crank arm, combined with high-performance materials, significantly improves the typical bicycle’s stiffness crank arm. However, the result holds limited validity due to the uncertain material properties of stainless steel 17–4 PH produced via 3D printing technology.

The article [2] is an example where the authors analysed the infill as a Triply Periodic Minimal Surface (TPMS) in the form of Gyroid, Schwarz D, and Schwarz P surfaces. They are minimal 3D surfaces, meaning a surface that locally minimises its area and has a mean curvature equal to zero at every point [2]. TPMS structures are periodic, continuous, non-self-intersecting, and infinite. Some of these surfaces are known enough to have names associated with them, such as Schwarz primitive, Schwarz diamond, Schwarz hexagonal, Schwarz crossed of parallels, Neovius, and Gyroid.

The Gyroid surface and shell lattices based on the Gyroid structure were discovered by Schoen [32]. The 3D model of the TPMS Gyroid surface geometry can be described using the following expression Equation (1).

(1) cos(ω·x)·sin(ω·z)+cos(ω·y)·sin(ω·x)+cos(ω·z)·sin(ω·y)=t

where *x*, and *y* are spatial coordinates, ω=2π/l, *l* is the unit cell’s length, and *t* is the level parameter of the isosurface, which can effectively control the relative density of the gyroid surface [32].

A Gyroid-based structure characterises low-stress concentrations due to zero mean curvature (no joints or discontinuities exist) [33]. Moreover, it has no planes of symmetry and no embedded straight lines, which is beneficial when filling complex geometry regions. Moreover, as shown in [34], the Gyroid infill is nearly isotropic, simplifying its implementation into design and optimisation workflows. Low anisotropy under compression of the Gyroid infill structure was also confirmed by [35]. Moreover, it was noted that the inner Gyroid structure and pattern are more relevant than the material used to build the structural part. In recent years, TPMS-based lattices have been proposed for various engineering applications: body implants, functionally graded structural lattices, heat exchangers and lightweight structures for mechanical components [36]. Furthermore, functional grading TPMS lattice can be used with a proper design to mimic the structure of natural systems [37,38]. Therefore, we assumed that the Gyroid lattice infill is an excellent choice for a 3D-printed infill of complex lightweight parts.

### 1.6. Crank Arm Design and Analysis

The crank arm design was a topic for various authors. They have used it for investigations with Finite Element Analysis (FEA) and structural optimisation [39,40,41,42]. Most of the time, the crank arms had a standard form known from the daily usage of bicycles. However, one can see an innovative crank arm design for additive manufacturing in [40] designed using topology optimisation to maximise stiffness at minimum mass by employing a finite element method. In the context of manufacturing, crack arms are primarily designed for forging and casting for purely economic reasons. However, for high-tech applications, they are made of carbon fibre composites. Materials for the crank arm are typical for lightweight structures, such as aluminium alloys, high-strength steel alloys, carbon fibre composites, and titanium. Nonetheless, bicycle crank arms are manufactured mainly of aluminium, fibre composite, and steel alloys. Material selection depends on bike types, targeted customer groups’ applications, and manufacturing costs. Due to the variety of materials, the stiffness-to-weight ratio of the cranks is highly variable.

## 2. Materials and Methods

### 2.1. Analysis Method

**Figure 2:**
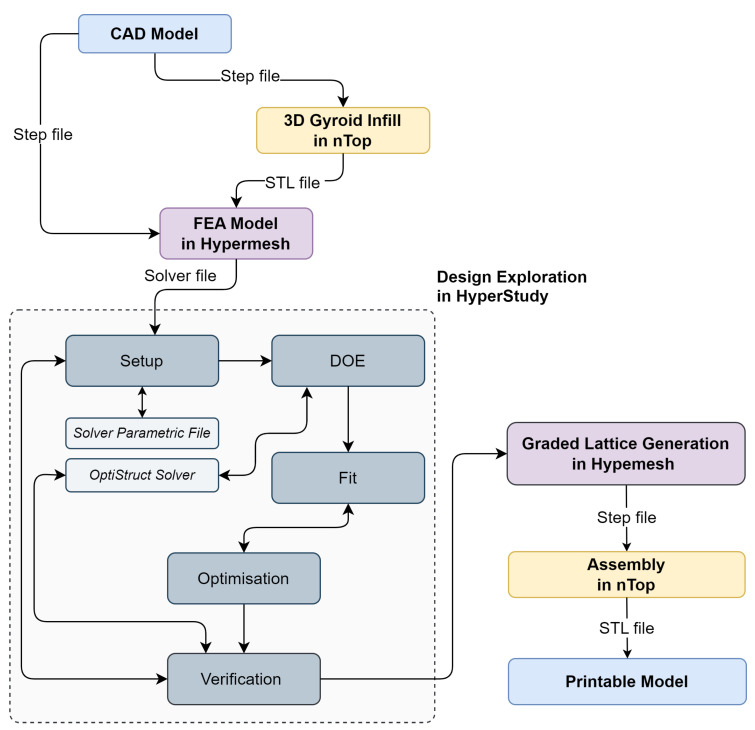
Flowchart of design method of the crank arm with the graded lattice infill.

Several tools to design 3D models exist, but not all can effectively create components with a lattice structure, mainly because of a lack of robust lattice generation methods and their proper representation in existing CAD systems. The manual process of building lattice models using existing CAD software is always very cumbersome. However, the software—nTopology (nTop), version 3.45.4. [43] can give some freedom in the design of the lattices, but exporting to CAD can be done only via mesh formats such as the Standard Triangle Language (STL) format, which is very inconvenient for further integration with other solids. We selected nTop software for the project since it has all the needed lattice generation capability and a sufficient interface to export the lattice model. However, due to the limitations of the simulation and optimisation routines implemented in the version of nTop software available at the time, our optimisation problem could not be performed in nTop. Therefore, Altair HyperMesh and HyperStudy software and programming were employed. The developed process is presented graphically as the flowchart in Figure 2. The design process started with creating a 3D CAD model; then, a Gyroid infill lattice was created employing nTop. Next, a shell body and the generated infill were served to build a finite element model in HyperMesh. After that, the FEA model was an input for the design exploration (DE) module, resulting in an optimum design of the lattice infill according to a defined optimisation problem. In the last step, the infill as the optimum graded lattice and the shell body were combined into one solid body using HyperMesh and nTop, resulting in an STL file of the print-ready model.

In order to compare the results of the optimised design with reference models, additional FEA models were analysed. So, four models were built using the same loads and boundary conditions as in the case study. These reference objects were a hollow crank arm model with no infill and two models from a previous publication [31], which have the exact dimensions as the optimised one but two different infills made of a Face-Centred Cubic (FCC) and (Re-entrant) strut-lattice and as well as the original Shimano FC-R450/453 geometry model made of aluminium.

### 2.2. Loading and Assessment Criteria

**Figure 3:**
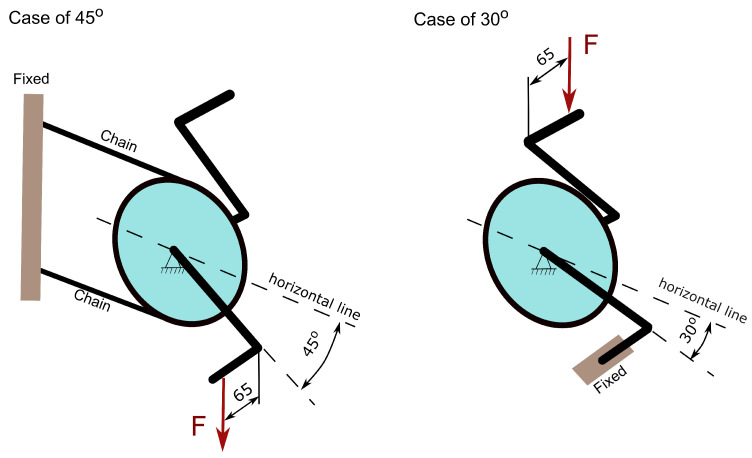
Load configurations defined by the standard [43].

Typical loading of the crank arms coming from pedalling is dominated by bending in two perpendicular planes and torsion. The loading is standardised by an ISO standard [44], and for design purposes, it is split into two loading groups—two fatigue load cases. Depending on the bicycle application, a vertical force of 1300 N or 1800 N is required by that standard with minimum test cycles of 100,000 or 50,000. The load is applied on a pedal with an offset of 65 mm from the outboard face of the crank arm. The direction of the crank arm with respect to the horizontal plane is 30${}^{\circ }$ or 45${}^{\circ }$ depending on the load case, as shown in Figure 3. The greatest fatigue force of 1800 N of the 45${}^{\circ }$ load case is defined for mountain and racing bicycles with cycling requirements of 50,000 and 100,000 cycles, respectively. For the 30${}^{\circ }$ load case, only a force of 1800 N is required with 50,000 cycles. The obligatory load values of the standard appear conservative compared to the test data shown in the article [45]—a 34-year-old healthy man can generate during 2 min on a bicycle power of 200 W with a rotational crank speed of 50 rpm. Therefore, he generates a torque of 38.2 Nm and considering the crank arm length of 170 mm, it translates to the force at the pedal of 225 N. The observed significant difference in the forces can be explained by the desire to ensure safety under all conditions, for example, when the force is applied dynamically by jumping on the pedal. In the presented work, only one fatigue load at 45${}^{\circ }$ (Figure 3) with a maximum force of 1800 N is analysed since it is the most severe case for this crank arm.

Detailed fatigue analysis can significantly complicate the whole design exploration process, causing the optimisation results to be incorrect because of a mesh-sensitive fatigue model and difficulty in result interpretation. Moreover, the fatigue endurance of a printed object significantly depends on the printing process parameters [17]. Consequently, a simplified assessment criterion was defined by a von Mises stress limit of stainless steel 17–4 PH of 360 MPa. The authors accepted that the chosen limit of von Mises stress is sufficient, considering the printed material strength [17] and the fact that the infill has extra small fillets of 0.35 mm that were not present in the FEA models because their implementation was practically impossible due to the complexity of the infill geometry. Nevertheless, fillets were applied in the stage of the infill assembly with the crank arm body in nTop software for printing preparation.

### 2.3. Infill as Surface-Lattice Structures

**Figure 4:**
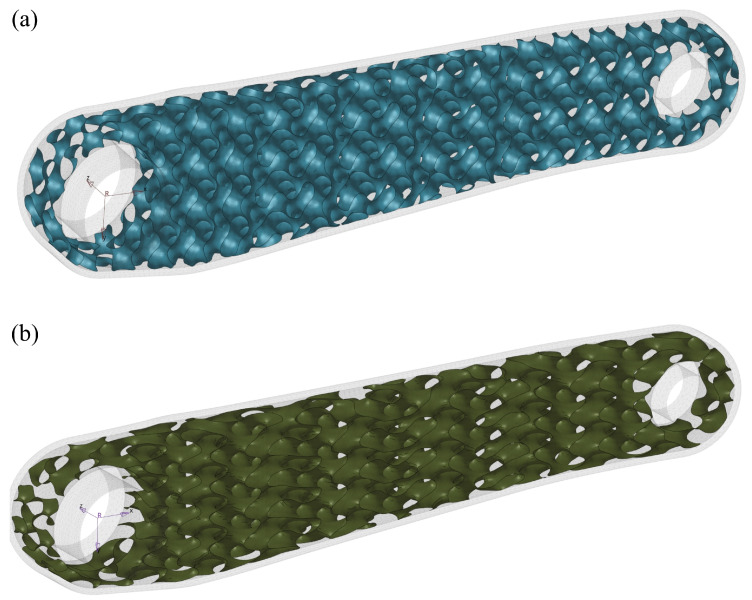
Internal shell-lattice as gyroid surface: (**a**) 10 × 10 × 10 mm lattice (X, Y, Z); (**b**) 18 × 8 × 10 mm lattice.

The selected lattice structure is the Gyroid TPMS with two different cell sizes: 10 × 10 × 10 mm (X, Y, Z) and 18 × 8 × 10 mm (Figure 4). The authors used the shell lattice with two unit cell sizes and varying thicknesses. As a result, optimal spatially varying shell lattice structure thickness was found to create the functionally graded infill. The cell size has been chosen to achieve a self-supporting infill structure and avoid closing the cell because of its size in the printed element. As a rule of thumb, an overhang that extends at a 45${}^{\circ }$ angle requires extra support to make it possible to print the structure. Reorientation of the printed object during printing can help to minimise the overhangs. Nevertheless, the proposed infill ensured a print without extra internal support. Unfortunately, the cell size selection limits possible design candidates for the infill.

**Figure 5:**
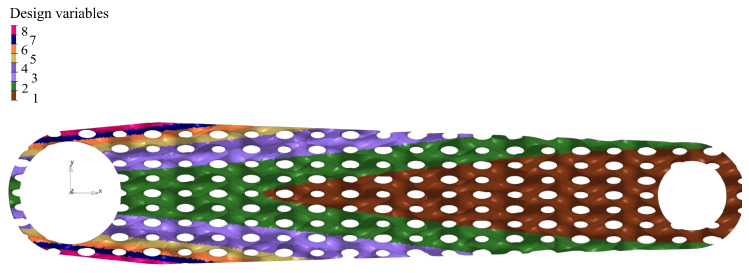
Initial 2D distribution of thickness of shell-lattice, design variables based on Equation (2) and then discretised in 8 groups.

The initial thickness pattern was identical for both lattice types. The pattern Equation (2) was selected based on the assumption that the crank arm must be symmetric and cantilever bending with torsion is the dominant loading. The defined pattern corresponds approximately to the stress distribution during pure bending. Generally, any thickness pattern is possible, but the choice limits potential solutions. The pattern distribution is displayed in Figure 5. The thickness variability has been limited to 8 design variables to minimise the computational time. The thickness parameters were obtained by dividing a thickness range specified by Equation (2) into eight groups, and the average group thickness was assigned to all group elements. Each variable had a continuous allowable range of variation of 0.5–1.5 mm. The minimum thickness was driven by the resolution of a 3D printer nozzle, while the maximum thickness was restricted by the desire to achieve a recognisable post-print structure of the lattice infill. Too small a cell size could cause a complete closing of the space in the cells in some parts of the infill where maximum thickness could be required.

The initial thickness distribution function shown in Figure 5 is defined as follows by the equation:

(2) $$T\left(x,y\right)=0.685-0.001\cdot x+\left(0.003-3.331\cdot 10^{-5}\cdot x+1.172\cdot 10^{-7}\cdot x^{2}\right)\cdot y^{2}\;$$

where *x*, and *y* are spatial coordinates and T(x,y)-thickness distribution in mm.

The crank arm design was chosen to be a shell body with a thickness of 1.6 mm with the presented functionally graded infill. The shell body thickness was selected, considering that a printer nozzle size for metal FFF technology can be 0.4–0.6 mm. A thinner wall can cause manufacturing problems with the proper representation of the wall thickness. A thicker wall can lead to an unwanted increase in the mass of the printed object.

### 2.4. Manufacturing Constraints

Manufacturing constraints can depend on the specific metal FFF technology. Currently, there are some available commercial solutions; the first one is Markforged with Metal X system [46], the second one is BASF with Ultrafuse material [47], the third one is Virtual Foundry [48], and the last one is Desktop Metal Studio System [49]. In the presented project, the Markforged Metal X system was used to produce a prototype part, and consequently, the following manufacturing constraints need to be considered in the design process:

- The material strength of the green parts determines the required support structure and limits the possible types of internal shell lattice structures.
- The sintering process limits the size of the printed parts and forces to use of support structures.
- The printed material and nozzle size determine the minimum thickness of a printed object.
- Metal X technology offers only some materials to print: 17–4 PH stainless steel, H13 tool steel, A2 tool steel, D2 tool steel, Inconel 625, and copper, whereas 316L stainless steel, titanium Ti6Al4V and aluminium are not yet available for this system.
- A substantial shrinkage after sintering reached approximately 20%, varying in a small range depending on part sizes.
- The material is characterised by high porosity because of the specific production process.
- Materials show strength anisotropy determined by a part orientation during printing.

For the presented project, the authors selected a filament developed by Markforged, stainless steel 17–4 PH (version 2), offering theoretically sufficient strength. As mentioned, bicycle crank arms are manufactured mainly of aluminium, fibre composite, and steel alloys. Therefore, the material selection does not seem unusual for the application.

### 2.5. 3D Printing, Debinding and Sintering Parameters

The crank arm made of 17–4 PH (version 2) stainless steel was manufactured using the Markforged Metal X system (printing time of 2 d 4 h) with the settings shown in Table 1.

The debinding and sintering processes were done in-house with the default parameters developed by Markforged [47]. The Wash-1, a solvent-based debinding system with Opteon SF-79 liquid, was utilised for debinding the green part for 1 d 8 h. For the sintering, Sinter 1, a tube furnace was employed to produce the 3D-printed crank arm.

### 2.6. Design Exploration

The design exploration methodology was employed to find the best possible configuration by modifying the design variables in a design space. The DE analysis was conducted using Altair HyperStudy software [50], the Altair OptiStruct [51]—a finite element solver, Multiobjective Optimisation (MOO) [52], and other auxiliary tools for data analysis. To begin this process, eight design variables—the lattice shell thickness pattern were selected, as shown in Figure 5, based on the distribution described by Equation (2). Those variables and other parameters were implemented in an input file of the OptiStruct solver via its parameterisation that allows an optimisation process. Once the finite element model and the design variables were established, design objectives were defined as the crank’s mass and its maximum displacement. We decided to use MOO, minimising the objectives with a design constraint defined as von Mises stress of 360 MPa. Since we had set it as multiobjective optimisation with two contradictory objectives to minimise the total mass and the maximum deformation, the design exploration gave not one but a set of optimal solutions.

Once the DE model was set up, a Design of Experiments (DOE) study was employed as prerequisite steps for an approximation stage, fitting a predictive mathematical model to the data to create a response surface model approximation (Figure 2). The DOE study’s objective was to distribute the design points uniformly in the design space to feed them into a fitting method to predict the model behaviour accurately. The Modified Extensible Lattice Sequences (MELS) method [53] was employed for the DOE studies. MELS is a quasi-random sequence designed to distribute the design points in space, minimising clumps and voids evenly, and is based on extensible lattice sequences [54]. The fitting process used the Fit Automatically Selected by Training (FAST) method [53], automatically building the best-fitting functions by testing all implemented methods. Then, instead of finite element analysis, that built approximation was utilised to shorten the optimisation process time, avoiding typical problems with limited computation resources. Following appropriate procedures, the multiobjective optimisation step was employed. The Global Response Search Method (GRSM) [53] was used for optimisation. The algorithm generates a few designs, including global sampling, to ensure the right balance of local and global search capability. The response surface is updated with the newly generated designs to improve the model fit. Afterwards, response surface-based optimisation is conducted. As a result of the described process, the non-dominated solutions (Pareto-optimal set) are determined. Here is where the entire analysis process ends, and the optimum solutions were selected, considering other factors that could not be considered during the DE process, such as manufacturing constraints.

### 2.7. Finite Element Model

**Figure 6:**
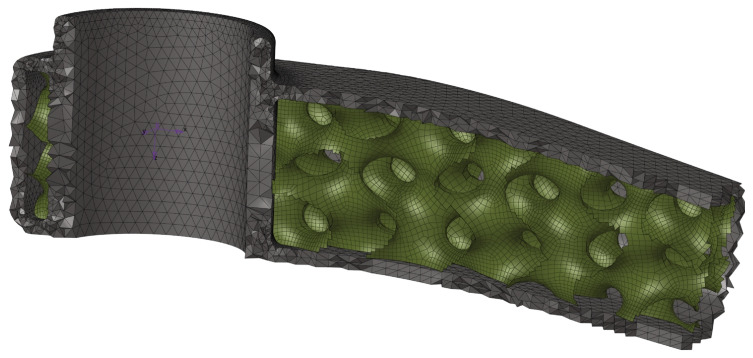
Analysed FE models with 10 × 10 × 10 mm (X, Y, Z) shell-lattice, view of solid and shell elements.

We built a crank arm finite element model using two elements: second-order 10-node tetrahedron elements and first-order shell quad elements (Figure 6). The OptiStruct solver was employed to analyse the developed model. Since the deformation of the part is expected to be small with stress below the yield strength, the FE analysis is linear, allowing for a shorter optimisation time. However, the model linearity does not limit the generality of the proposed method in any way. The shell model of the internal lattice infill allowed us to parametrise the models required for the design exploration phase. Contact elements connect the external solid elements with the shell elements of the infill (Figure 6). The initial lattice thickness distribution was defined, as illustrated in Figure 5, applying eight design variables (shell element thickness). For that purpose, a TCL/TK script was developed that assigns a particular thickness to the shell elements with a defined number of the variables based on Equation (2). The reduced number of parameters contributed to the simplicity when optimising and generating a final STL model. However, increasing the variables at the cost of additional modelling complexity is possible.

#### 2.7.1. Load and Boundary Conditions

**Figure 7:**
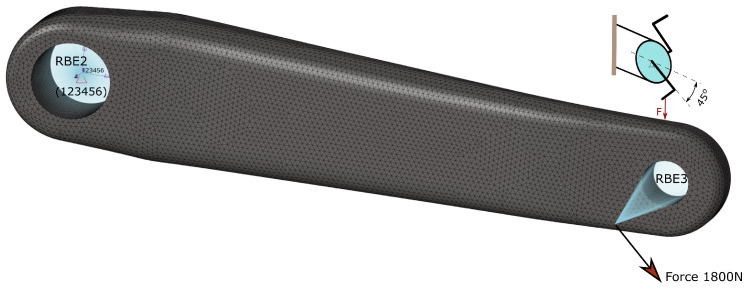
Load and boundary conditions.

As we described earlier, only the load case of the fatigue at the crank arm location of 45${}^{\circ }$, with a maximum force of 1800 N, is analysed since it is the most severe case for the designed crank arm. The force was applied using the distributing type 3 rigid body element (RBE3), and the crank arm was fixed using the RBE2 element, as stated in Figure 7. The drive (independent) node of the RBE2 element was created in the centroid of the large hole used to fix the crank arm onto the shaft. All degrees of freedom of that node were fixed.

The force offset of 65 mm from the outboard face of the crank arm was used to simulate a realistic load condition; the force was assumed to be applied to the middle of a pedal.

#### 2.7.2. Contact

The solid and shell elements are connected via contact elements; all degrees of freedom of nodes of both element types are bounded via a contact penalty algorithm. The Freeze contact interface is used, which enforces zero relative motion on the contact surface, and the rotations at the slave node are matched to the rotations of the master patch. The Freeze contact type is predefined in the OptiStruct solver as one of the offered contact types.

#### 2.7.3. Materials of Models

The mechanical properties of 17–4 PH (version 1) stainless steel as printed are presented in Table 2. The data come from the Markforged document [55] and the articles [17,56], where the properties have been experimentally verified, and as we can see in these publications, the 17–4 PH steel is very brittle as printed with varying strength caused by material defects. In the article [17], the authors argue that applying the metal FFF technology for structural parts is risky. Even so, the choice of FFF metal technology for the current project is enforced, as it allows parts to be manufactured without trapped metal powder inside the infill. The technology still offers a high potential for further development.

Seeing the large spread of material properties and existing anisotropy of material caused by the printing, we decided to select the properties shown in Table 3 for our research and used a linear isotropic material. Additionally, one of the reference models was the original Shimano FC-R450/453, made of a precipitation-hardened aluminium alloy, and its properties are listed in Table 3.

## 3. Results

### 3.1. Design Exploration

**Figure 8:**
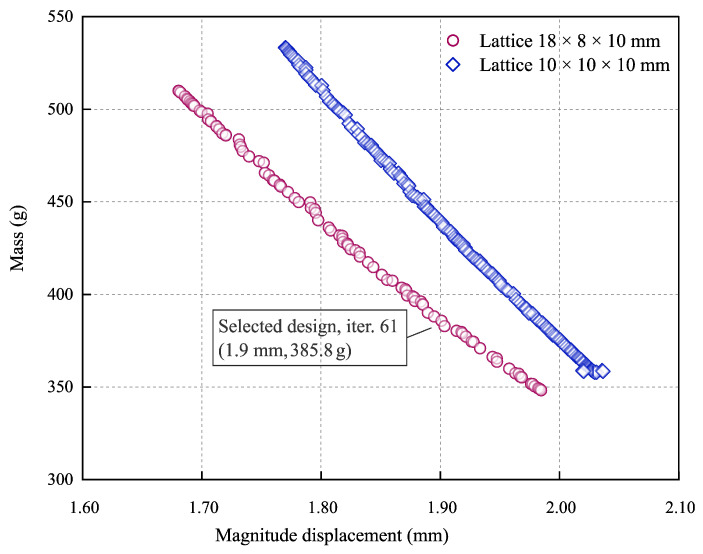
Pareto-optimal set of solutions.

The output of the design exploration step is shown in Figure 8. A Pareto plot shows the sets of optimum designs for the defined constraints and objectives. The figure compares the results obtained from the analysis of the two models analysed of the shell-lattice types. The two curves obtained are linear, with Pearson’s R coefficient being adequately −0.9986 and −0.9995 for 18 × 8 × 10 mm and 10 × 10 × 10 mm curves, and offset from each other. The curve for the 18 × 8 × 10 mm lattice is shifted to the left of the 10 × 10 × 10 mm lattice curve. The figure shows that the solution with lattice 18 × 8 × 10 mm performs better regarding the stiffness-to-mass ratio. The difference between both lattice types is significant and reduces with the mass decrease since both curves have a different slope. The curve slope of the 18 × 8 × 10 mm lattice is smaller than the second lattice. Looking at Figure 8, it is apparent that there are many different possible solutions; however, the printability without internal support and the print resolution limit the possible design candidates.

**Figure 9:**
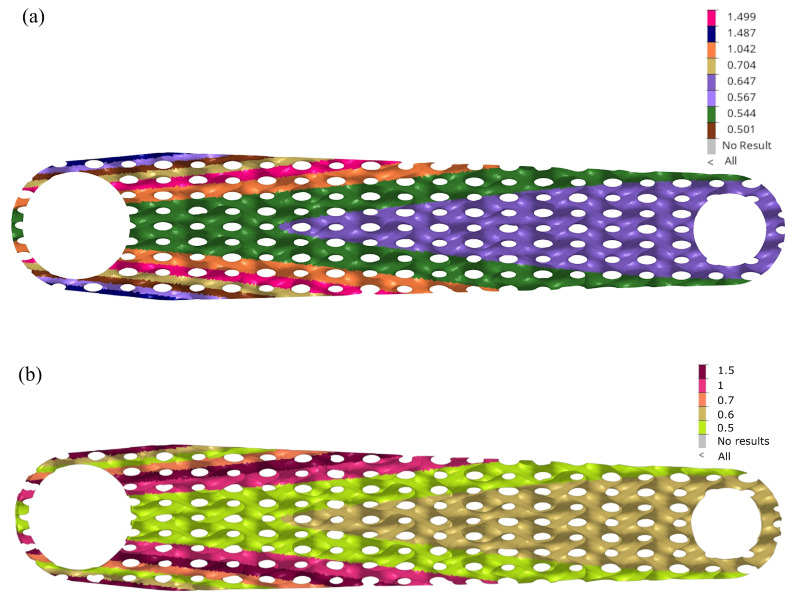
Thickness distribution of shell-lattice of infill: (**a**) thickness of selected optimum design iter. 61; (**b**) thickness defined for printed prototypes.

The minimum mass of 348.3 g was obtained for the constant thickness of the lattice of 0.5 mm for the lattice 18 × 8 × 10 mm with a maximum displacement of 1.985 mm. Unfortunately, this solution is not the best for manufacturing reasons and print quality; as explained, 0.5 mm thickness is difficult to achieve for the infill during printing. Including the part oversizing because of the post-sintering shrinkage, the wall of 0.5 mm needs to be printed as 0.6 mm. The nozzle size utilised in the experiment was 0.4 mm. Consequently, printing lines with thicknesses that deviate from multiples of the standard line width of 0.4 mm, as defined by the Markforged preprocessor, deteriorate part quality due to poor material connectivity at the point where lines merge. As a result, selecting a structure with a slightly thicker minimum wall thickness to mitigate these line interface issues is a more prudent approach. This would also increase rigidity and facilitate the manufacturing process of the arm. Thus, to prototype the arm, the authors choose the design that is marked in Figure 8—iteration 61. The selected design has a mass of 385.8 g with a resulting displacement of 1.9 mm. We showed the thickness distribution of the selected solution (iter. 61) in Figure 9a, and in this design candidate, the region of the critical thickness of 0.5 mm is greatly reduced. Considering technological limitations, this distribution was slightly adjusted to the printing prototypes (Figure 9b).

The general thickness distribution is not surprising—it follows approximately a bending stress map in a cantilever beam. However, a specific local perturbation in the distribution is unexpected around the large hole: a sudden decrease in thickness in the lattice’s outer layers and a renewed increase.

### 3.2. Optimum Solution

**Figure 10:**
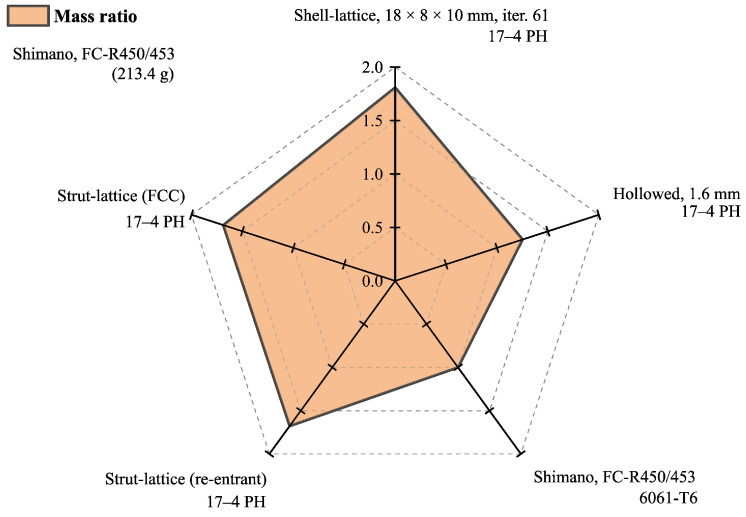
Mass comparison between designs.

Comparing the mass of the analysed crank arms results in Figure 10 and Table 4, the chosen design with shell lattice has 1.81 times greater mass than the reference design and slightly more than the crank arm with the strut-lattices having that ratio of 1.67. It should be remembered that the reference arm (Shimano FC-R450/543) is made of aluminium, and the others are made of stainless steel. Therefore, the obtained ratio of 1.81 is a reasonably good result compared to the specific density ratio of 2.8 between steel and aluminium. The authors added the mass of the hollowed arm with a wall thickness of 1.6 mm, having a ratio of 1.25. However, it must be remembered that the hollowed construction does not meet the criteria for maximum permissible von Mises stresses of 360 MPa.

**Figure 11:**
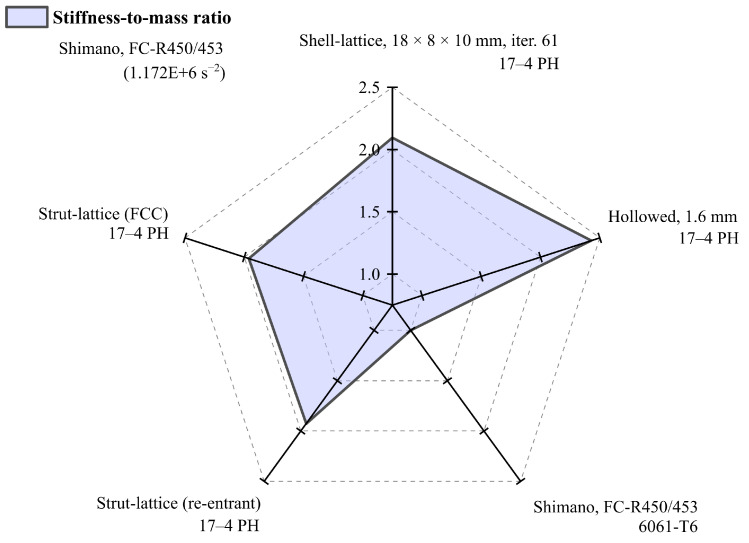
Stiffness-to-mass ratio comparison between designs.

More exciting results are shown in Figure 11 and Table 4, where the stiffness-to-mass ratio is presented for the same structures. Here, we see that the developed arm with the surface lattice is by far the best in this category, which also respects the stress restriction. The ratio reaches a value of 2.10 compared to the design with the strut-lattices of 1.67, including the reference design of 1. The best overall solution for 2.43 is the hollow design, but it should be remembered that it is excluded because of unacceptable stress levels.

**Figure 12:**
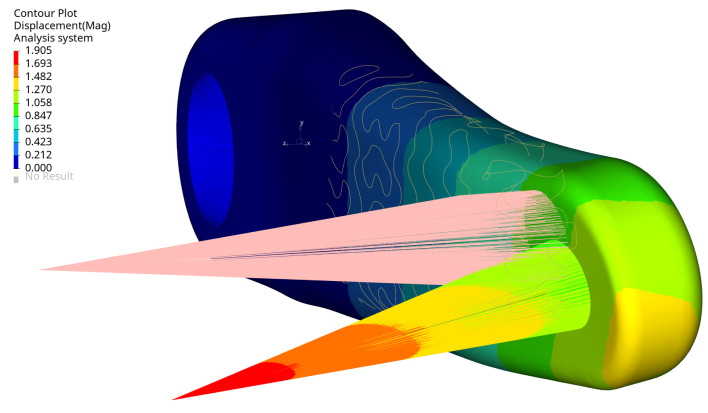
Magnitude of displacements (mm) selected optimal design, iter. 61.

Figure 12 presents the magnitude of displacement of the selected arm design under a defined load, as the evidence shows that the load is dominated by bending in two planes and torsion. For the structural assessment, the maximum displacement of the node at the pedal centre (load application point) was considered.

**Figure 13:**
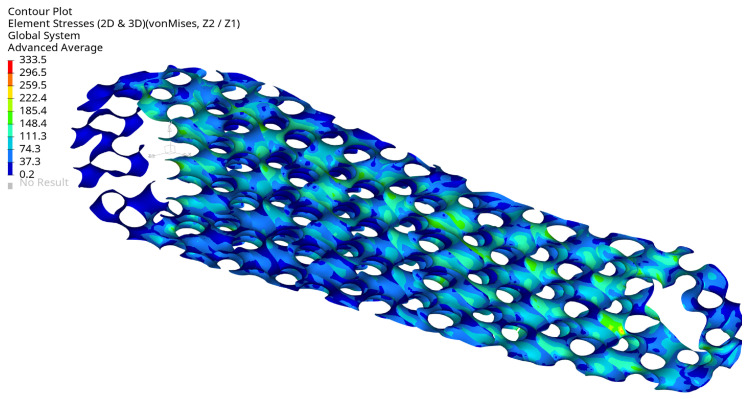
Maximum von Mises stress (MPa) for selected optimal design at maximum load, view 1, iter. 61.

**Figure 14:**
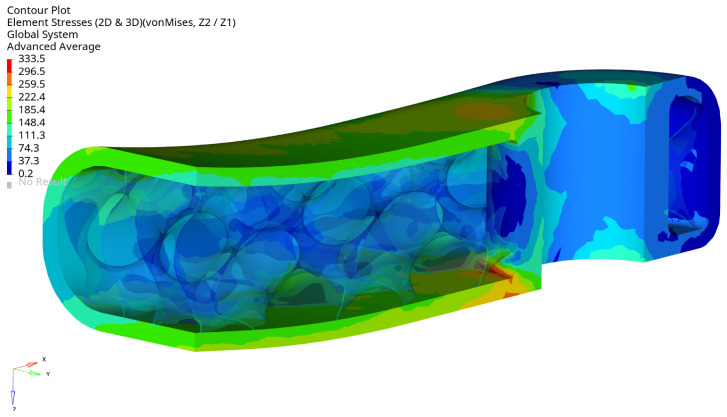
Maximum von Mises stress (MPa) for selected optimal design at maximum load, view 2, iter. 61.

**Figure 15:**
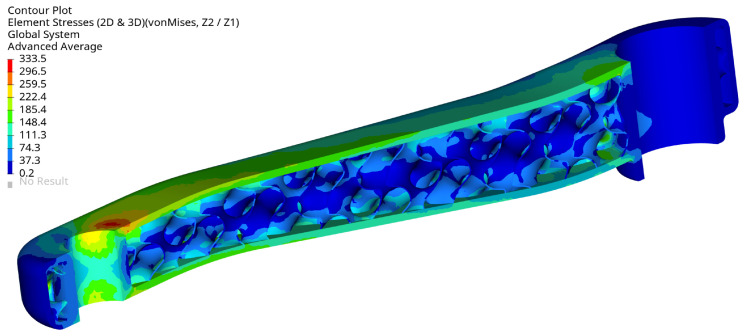
Maximum von Mises stress (MPa) for selected optimal design at maximum load, view 3, iter 61.

Figure 13 displays the von Mises stress in the crank arm’s internal shell lattice for the selected optimal configuration. As can be seen, the stress level is below the defined stress limit of 360 MPa. The results are plotted from the shell elements’ external layers (top and bottom). The maximum stress is located around the small hole in the crank arm. There, the load is applied through the RBE3 element. The maximum stress position is not surprising; they should typically be expected around the fixation and the load location. This effect can also be seen in Figure 14 and Figure 15 on the arm’s external surface close to the hole used to assemble a pedal. That stress concentration shows the level of von Mises stresses below 333 MPa.

Figure 13 shows that the maximum stress of 333.5 MPa is in the arm’s shell part inside the cavity on the radius. The stress level is acceptable, but it is evident that local geometry modifications can reduce the stress in future investigations.

**Figure 16:**
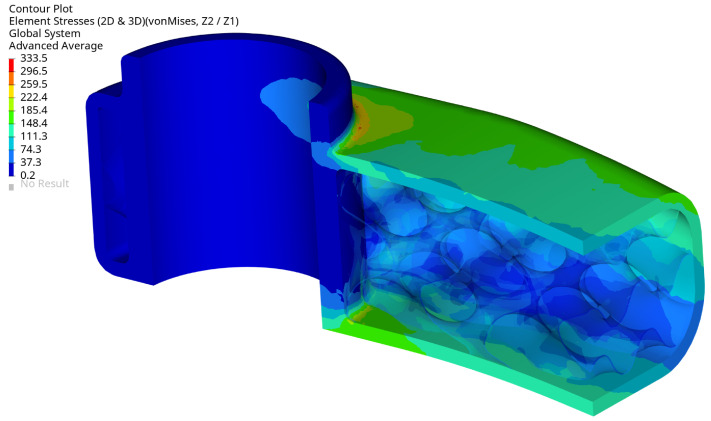
Maximum von Mises stress (MPa) for selected optimal design at maximum load, view 4, iter. 61.

The arm’s fixation looks very well designed, the stress level is relatively low, and the design does not need to be modified. The stress level is below 333.5 MPa, and the maximum is located in the radius of the external surface Figure 16. The maximum stress localisation is fully explainable and expected due to how it is fixed and the load type. In reality, lower stresses are expected because the used boundary conditions are simplified, resulting in stiffening the places where the loads have been applied. That effect translates into higher stresses and is created directly by the RBE2 element, which adds locally infinite stiffness to a structure.

### 3.3. Prototype

**Figure 17:**
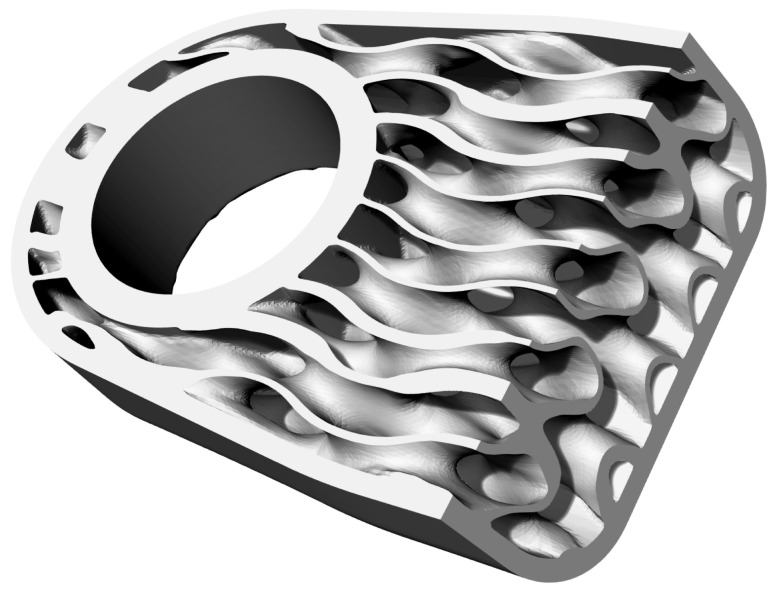
Internal structures, iter. 61, STL model (part of the whole model).

Complex geometry requires many elements to represent the print model properly; therefore, the size of the generated STL file (inter. 61) was huge, about 600 MB. Finally, it was possible to handle the file without significant difficulties. A part of the generated STL model is shown in Figure 17; it reflects the complex internal geometry of the developed infill.

**Figure 18:**
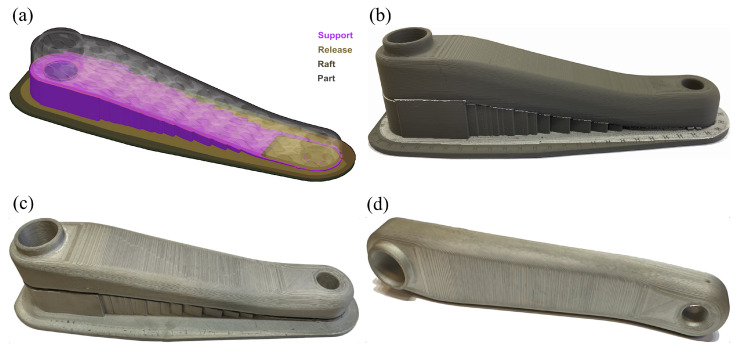
Prototype of the crank arm, iter. 61: (**a**) model with support and raft structures; (**b**) green part; (**c**) sintered part with support structure; (**d**) sintered part.

The sintered crank arm (Figure 18d) had a mass of 343.8 g and no visible cracks on its external surfaces. The green part and the sintered one had a typical surface roughness determined by layer-by-layer material deposition of the metal FFF technology, as shown in Figure 18. Interestingly, the measured mass of 343.8 g of the crank arm represented only 89% of the 3D model’s mass of 385.8 g, and an explanation of this will be provided later in the article.

**Figure 19:**
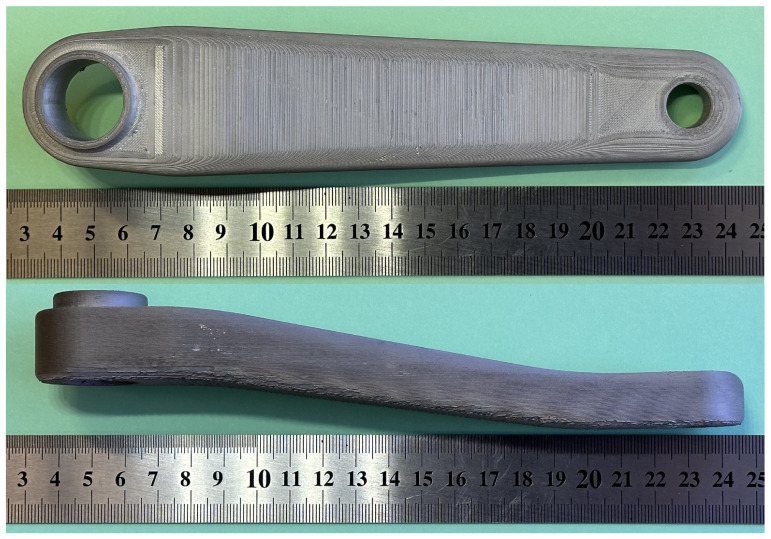
Views of the printed and sintered crank arm (iter. 61).

The printing and sintering process achieved the crank arm’s desired external dimensions, as shown in Figure 19. The external surface of the part has a typical metal FFF technology texture caused by the distribution of material layers while printing.

**Figure 20:**
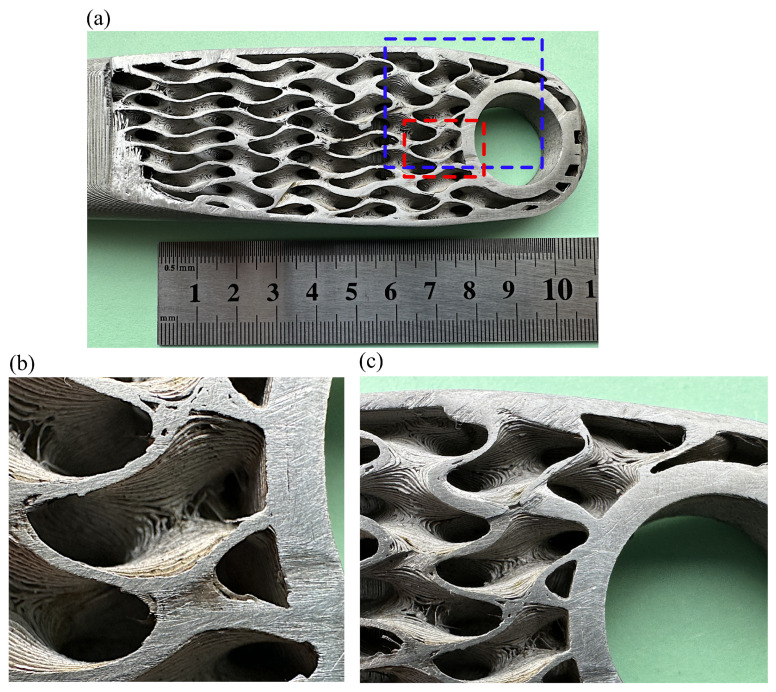
Views of the sintered cut crank arm (iter. 61) showing the internal structure: (**a**) whole cut section; (**b**) view of the infill marked by the red rectangle, (**c**) view of the infill marked by the blue rectangle.

Additionally, the infill structures showed the same typical texture of metal FFF parts. However, the infill was not represented entirely correctly; Figure 20a–c shows that unsintered and missing layers caused gaps in the infill, occurring randomly throughout its volume.

## 4. Discussion

It was hypothesised that the functionally graded internal shell lattice could be implemented in real mechanical parts and that the applications could benefit from increased stiffness-to-mass ratios. The literature shows that many works are devoted to specimens rather than geometrically complex parts. The presented work shows that it is feasible to design the functionally graded lattice as the infill in the crank arm using the metal FFF technology and existing engineering tools. A novel design and analysis method is proposed. In detail, the authors show how to efficiently represent the crank arm’s geometry with the functionally graded infill and how to search the design space for the optimum design. Finally, the optimum configuration was selected considering the manufacturing requirements from the sets of optimum designs for the defined constraints and objectives represented by the Pareto plot in Figure 8. The results show that a satisfactory outcome regarding the stiffness-to-mass ratio is possible. In the presented work, that ratio is much better than the existing reference design (Table 4). A percentage increase of 109.6% was achieved, although the lattice cell was chosen to meet the manufacturing requirements (overhangs and visible lattice structure after sintering). Theoretically, there is room for improvement by selecting a more appropriate cell grid and thickness distribution, but this requires developing a new sophisticated selection strategy that must include aspects of a chosen manufacturing method.

The authors believe the chosen stress limit of 360 MPa for optimisation is appropriate for the given loads and considering the project’s objectives. The structural performance of the optimised crank arm was accomplished based on the finite element method for the simplified model without the small fillets in the infill, which makes the assessment more conservative. As the developed finite element model showed, a theoretical assessment of the structural performance of the mechanical elements, such as a crank arm with the graded internal infill, is feasible. However, because of the considerable strength variability caused by internal material defects shown in work [17], there should be no illusion that metal FFF technology can currently be applied to functional parts. It is hoped that further development into improving the material strength of parts produced by the metal FFF printing process will enable their application. It is particularly important when applied to crank arms, as failures due to fatigue fractures of crank arms can occur in bicycles [57,58]. It is crucial to ensure a safe design, as failure can cause serious injury to the cyclist. Thus, it can be concluded that the designed crank arm currently does not fulfil functional requirements because of safety concerns.

The most important result of the work was the original elaborated method of the structure’s design with functionally graded lattice structures. The proposed method can adapt to any structure, boundary conditions, and manufacturing constraints. Design exploration and finite element analysis together can be excellent tools for that problem, as shown in the article. Unluckily, the procedure is required to assume a pattern of the lattice cell distribution; however, it enables us to consider the manufacturing constraints. Additionally, the initial distribution of the lattice thicknesses is recommended to reduce the number of design variables to accelerate optimisation. A dream method would be a design exploration procedure containing a mechanism that gives a graded distribution of grid cells with variable thickness with no initial constraints. It is conceivable that a field defined by an equation, point cloud, or a stress gradient could guide the grid cell size distribution and variable thickness and consider manufacturing constraints. The authors believe it is currently impossible or unnecessary to create such a method; that is confirmed because no one has shown such a working solution for a complex three-dimensional part.

The following important outcome of the project is testing the limits of the metal FFF additive technology. Unexpectedly, the most complicated part of the project was additive manufacturing. Although printing technology opens up space to create complex structures, there are still areas where further development is necessary. The crank arm’s example with the complex infill shows the technical limitations of the technology. Here, we can start pointing out the obstacles. Firstly, slicing software cannot always cope with huge data sets. As shown in the project, the STL file format forced the handling of huge model files of hundreds of megabytes for complex geometry parts. Secondly, slicing software is often limiting in its ability to modify support structures manually. For instance, it auto-generates a predefined support structure that needs to be manually changed, which can be very difficult for complex geometry. Thirdly, the metal FFF technology limits printing thin-walled infill structures due to the current material properties and the nozzle size.

An example can be seen in Figure 20, where the defects are observed. Most likely, the faults were caused by the localised material collapse while printing because of the filament properties and insufficient support of the complex thin-walled infill. The last main obstacle is the maximum size of printed objects, often limited to 160 × 130 × 300 mm by Markforged, Metal-X Sinter-2 oven. That constraint is caused mainly by sintering process capabilities. Manufacturing larger objects is workable, but problems during the sintering process are expected. As shown in the paper, the printing time is also an issue because it can be long for parts with thin-walled internal structures reaching dozens of hours. As an example of the difficulties, it is noteworthy that it took the authors about a year to print the prototype shown in Figure 19, and only the latest version of the slicer software Eiger.io [59] and version 2 of 17–4 PH filament made printing possible with shown quality in Figure 20.

As was mentioned, we noticed a significant variation between the masses of the model and the actual printed and sintered parts by 11%. The pure porosity present in the sintered part cannot explain its magnitude. The maximum observed porosity in the metal FFF technology is 6.5% based on the review presented in [17]. Therefore, additional factors must contribute to the mass difference. So, the discrepancy is caused by the inappropriate geometric representation of the internal lattice in the prototype (Figure 20). Thus, the shown manufacturing faults contribute to the observed difference. Unfortunately, a precise measurement of the infill geometry in the sintered part is unrealistic, so no proof of measurement results can be provided.

Root causes of the observed manufacturing faults of the infill structure are determined by insufficient infill support, the minimum thickness of the infill, and the filament material strength. The orientation of the infill lattice and its size can be optimised using, for example, multi-objective optimisation similar to those proposed by the authors [60], who successfully showed the method to determine the build orientation in the SLM process automatically. The other parameters required, unfortunately, more profound modifications of metal FFF technology. Thus, it can be concluded that manufacturing the part with a functionally graded infill is feasible in the metal FFF technology; however, the selections of the structure infill type and its minimum thickness are crucial for a successful print.

## 5. Conclusions

This study aimed to demonstrate the feasibility of designing and manufacturing parts with optimised internal infills in the form of a functionally graded shell lattice structure using metal FFF technology. The results indicate that this approach significantly enhances the structural lightness of the fabricated part. The study suggests that using functionally graded lattices based on shell structures as the infill can significantly improve the stiffness-to-mass ratio within a defined stress limit. In this case, a percentage increase of 109.6% was achieved. This supports the idea that mimicking natural materials with functionally graded lattice structures can benefit parts. The presented analysis method shows that it is feasible to identify an optimal design of the graded infill among many viable options with manufacturing constraints. A detailed structural assessment method for the optimised crank arm was also presented. This study proves that the existing technology software, analysis methods, and hardware are developed satisfactorily to support the design process. The evidence suggests that designing the structures with functionally graded lattices is feasible for complex parts, and an example of the methodology was shown. Nonetheless, further advancements in metal FFF technology are necessary because several limitations were encountered while implementing the functionally graded lattice in the design. The restrictions concern additive manufacturing and its technical limitations in software and hardware. The most critical ones concerning 3D printing and sintering complex infill parts are the minimum wall thickness, the maximum part size, and filament materials.

More broadly, development is needed to improve three elements in the future:

- Firstly, work is required to improve the metal FFF technology for printing thin-walled internal structures, with issues relating to nozzle size, printing parameters and printer control algorithms.
- Secondly, improvements are required in metallic filament materials for the metal FFF technology to enhance printability and reduction of post-sintered internal defects, thereby increasing the strength.
- Thirdly, it is recommended to develop methods for generating field-controlled lattice structures and integration in CAD systems that enhance design optimisation and exploration, opening up space for new types of high-performance parts.

Due to the significant strength variability caused by internal material defects shown in the article [17] and possible manufacturing imperfections shown in the presented work, we do not recommend using metal FFF technology for functional parts at this time.

The primary study’s strength is the presentation of the in-depth design process of those structures supported by a shown case study of the crank arm and an identification of the current boundaries of metal FFF technology. Thus, the work contributes to the existing design knowledge for additive manufacturing technology by providing detailed design, simulation, and manufacturing methods.

## Figures and Tables

**Table 1:** Printing settings for the Markforged 17–4 PH (version 2) filament.

Printing Parameters	Value
Nozzle size, mm	0.4
Layer height, mm	0.125
Print bed temperature, °C	115
Metal hotend temperature, °C	220
Chamber temperature, °C	48
Oversizing factors: X, Y, Z, %	19.5, 19.5, 20

**Table 2:** Material properties reported in the literature for 17–4 PH (version 1) as sintered.

Parameters	[16]	[54]	[55]
Print direction	ZX (Upright)	XY (Flat)	XZ (On Edge)
Young’s modulus, GPa	142	140	189
Poisson’ ratio	-	0.272	-
Tensile strength, MPa	496	1050	815
Yield strength, MPa	441	800	650
Elongation at break, %	0.4	5	0.86
Density, g/cm^3^	-	7.44	-
Hardness	261 HB	30 HRC	-

**Table 3:** Material properties used in the analysis.

Parameters	Stainless Steel 17–4 PH (As-Sintered)	Aluminium 6061–T6
Young’s modulus, GPa	170	69
Poisson’ ratio	0.27	0.33
Stress limit, MPa	360	-
Density, g/cm^3^	7.44	2.7

**Table 4:** Results of analysed designs.

Design	Material	Mass, g	Displacement, mm	Stiffness, N/mm	Stiffness-to-Mass, $10^{6}\cdot \left(1/s^{2}\right)$
Shimano FC-R450/453	6061–T6	213.4	7.20	250.2	1.172
Strut-lattice (re-entrant)	17–4 PH	357.0	2.61	689.7	1.927
Strut-lattice (FCC)	17–4 PH	360.0	2.55	705.9	1.961
18 × 8 × 10 mm (Iter. 61)	17–4 PH	385.8 *	1.90	947.4	2.456
Hollowed, 1.6 mm	17–4 PH	267.3	2.37	760.5	2.845

* The printed crank arm had 343.8 g.

## Data Availability

All data used to support the findings of this study are available from the corresponding author upon request.

## Abbreviations

The following abbreviations are used in this manuscript:

AM	Additive Manufacturing
CAD	Computer-Aided Design
DE	Design Exploration
DOE	Design of Experiments
DSE	Design Space Exploration
FCC	Face-Centred Cubic
FDM	Fused Deposition Modelling
FEA	Finite Element Analysis
FFF	Fused Filament Fabrication
FGM	Functionally Graded Materials
FGS	Functionally Graded Structures
GRSM	Global Response Search Method
ISO	International Organisation for Standardisation
MELS	Modified Extensible Lattice Sequences
MEX	Metal Extrusion Method
MOO	Multiobjective Optimisation
nTop	nTopology
PBF	Powder Bed Fusion
SLS	Selective Laser Sintering
TPMS	Triply Periodic Minimal Surface
